# Genome-Wide Identification of AMT2-Type Ammonium Transporters Reveal That CsAMT2.2 and CsAMT2.3 Potentially Regulate NH_4_^+^ Absorption among Three Different Cultivars of *Camellia sinensis*

**DOI:** 10.3390/ijms232415661

**Published:** 2022-12-10

**Authors:** Wenjing Zhang, Lin Lin, Tao Wang, Mingjie Chen, Bo Song, Weijiang Sun

**Affiliations:** 1 College of Horticulture, Fujian Agriculture and Forestry University, Fuzhou 350000, China; 2 Ministerial and Provincial Joint Innovation Centre for Safety Production of Cross-Strait Crops, Fujian Agriculture and Forestry University, Fuzhou 350000, China; 3 Engineering Technology and Research Center of Fujian Tea Industry, Fuzhou 350000, China; 4 FAFU-UCR Joint Center/Horticultural Plant Biology and Metabolomics Center, Haixia Institute of Science and Technology, Fujian Agriculture and Forestry University, Fuzhou 350000, China

**Keywords:** tea plant, AMT2, NH_4_^+^ uptake kinetics, expression analysis, gene function identification

## Abstract

Ammonium (NH_4_^+^), as a major inorganic source of nitrogen (N) for tea plant growth, is transported and distributed across membranes by the proteins of ammonium transporters (AMTs). However, the AMT2-type AMTs from tea plants remain poorly understood. In this study, five *CsAMT2* subfamily genes were identified in tea plant genomes, and their full-length coding sequences (CDS) were isolated from roots. Then, a NH_4_^+^ uptake kinetic comparison of Fudingdabaicha (FD), Huangdan (HD), and Maoxie (MX) showed that FD was a high N efficiency (HNE) cultivar that had a wide range of adaptability to NH_4_^+^, HD was a high N efficiency under high N conditions (HNEH) cultivar, in which it was easy to obtain higher yield in a high N environment, and MX was a high N efficiency under low N conditions (HNEL) cultivar, which had a higher affinity for NH_4_^+^ than the other two. Tissue-specific expression analysis suggested that *CsAMT2.2* and *CsAMT2.3* were highly expressed in the roots, indicating that these two members may be unique in the *CsAMT2* subfamily. This is further supported by our findings from the temporal expression profiles in the roots among these three different N adaptation cultivars. Expression levels of *CsAMT2.2* and *CsAMT2.3* in FD and HD were upregulated by a short time (2 h) under high NH_4_^+^ treatment, while under low NH_4_^+^ treatment, *CsAMT2.2* and *CsAMT2.3* were highly expressed at 0 h and 2 h in the HNEL-type cultivar—MX. Furthermore, the functional analysis illustrated that *CsAMT2.2* and *CsAMT2.3* could make a functional complementation of NH_4_^+^-defective mutant yeast cells at low NH_4_^+^ levels, and the transport efficiency of *CsAMT2.3* was higher than that of *CsAMT2.2*. Thus, we concluded that *CsAMT2.2* and *CsAMT2.3* might play roles in controlling the NH_4_^+^ uptake from the soil to the roots. These results will further the understanding of the NH_4_^+^ signal networks of AMT2-type proteins in tea plants.

## 1. Introduction

The tea plant (*Camellia sinensis* (L.) O. Ktze.) is a significant evergreen crop for leaf-use, and thus, N (nitrogen) is an essential nutrient in the process of its growth and development [1]. Appropriate N increases the content of leaves’ chlorophyll, promotes the biosynthesis of amino acids [2], balances the lipid metabolism source compounds of aroma [3], and facilitates the accumulation of most flavanol glycosides [4], thus improving tea products’ quality. At present, various tea plant cultivars are extensively grown in more than 30 countries across the world, and the plantation and production of tea could increase the incomes of local smallholders and promote local economic development [5]. However, in pursuit of high yield and high quality, the excessive application of N fertilizers is still common in tea gardens. According to the statistics, over 30% of the excessive application of N in tea garden areas is in China [6]. Such a phenomenon leads to higher costs, soil acidification, and greenhouse gas emissions including ammonia, nitrous oxides, methane, carbon dioxide, etc., which is not conducive to the green and sustainable development of tea production [7,8]. Therefore, a comprehensive understanding of N uptake characteristics among different cultivars of tea plants and their intrinsic molecular regulatory mechanisms is a guideline to optimize cultivation technologies to increase energy efficiency and reduce environmental pollution [9].

Nitrate (NO_3_^−^–N) and ammonium (NH_4_^+^–N) are two main inorganic N forms that can be utilized by plants in agricultural soils [10]. Unlike other plants, tea plants are well adapted to NH_4_^+^–N as the sole N source because of their NH_4_^+^ influx rates and assimilation capacities are much higher than for NO_3_^−^, leading to increases in theanine, glutamine, glutamic acid, and aspartic acid in both the leaves and roots with NH_4_^+^–N supply [1,11,12]. The transportation of NH_4_^+^ by the roots from the soil is the start of ammonia assimilation and the foundational step for N utilization [13]. In a wide range of plants, NH_4_^+^ transport across membranes is mediated by the proteins of the ammonium transporter/methylammonium permease (AMT/MEP) family, which can be divided into two categories, AMT1 and AMT2. All these proteins differ in their molecular structure, transported substrates, and affinity for the substrates [14,15]. In *Arabidopsis*, there are five members in the *AtAMT1s* subfamily. *AtAMT1.4* is a pollen-specific protein that transfers NH_4_^+^ across the pollen plasma membrane [16]. The other four AMT1-type members are all high-affinity transporters with different substrate affinity concentrations, and their specific spatial organization enables them to participate in NH_4_^+^ transport together through apoplasmic and symplasmic pathways [17]. The structure of AMT2-type transporters is similar to the AMTs in prokaryotes and they belong to the MEP subfamily [18]. There is only one AMT2-type transporter—*AtAMT2.1* in *Arabidopsis* [19]. However, in rice (*Oryza sativa*), there are at least ten AMT2-type homologs [20]. They are divided into multiple clusters—AMT2, AMT3, AMT4, and AMT5, and all belong to the AMT2 subfamily [21,22].

Several AMT2 subfamilies have been identified in other plant species, including *Populus trichocarpa* (PtrAMT2.1, PtrAMT2.2, PtrAMT3.1) [23], *Pyrus betulaefolia* (PbAMT2, PbAMT3) [24], *Lotus japonicus* (LjAMT2.1, LjAMT2.2) [25,26], *Brassica juncea* (BjAMT2) [27], and *Malus hupehensis* (MhAMT4.2, MhAMT4.3) [28]. In contrast to AMT1, the expression patterns of AMT2-type genes display more various features [23,24,29], and the fluctuation of their transcription levels depend on the external N nutritional status and the plant’s own N assimilates [21,22]. In rice, the expressions of some AMT2 transporters were dissimilar in cultivars with different N requirements [30,31,32]. Furthermore, the AMT2 subfamilies are involved in multiple physiological processes of N uptake and utilization. AtAMT2.1 plays a pivotal role in the NH_4_^+^ translocation from root to shoot of *Arabidopsis* [33]. LjAMT2.2 [26] (*Lotus japonicus*), MtAMT2.3 [34] (*Medicago truncatula*), and SbAMT3.1 [35] (*Sorghum bicolor*) are conducive to NH_4_^+^ transfer during arbuscular mycorrhizal symbiosis, and TaAMT2.3a (*Triticum aestivum*) easily uptakes N by the rust fungi infective pathogen to aggravate wheat disease [36].

Currently, only one AMT2-type gene—*CsAMT3.1*—has been isolated in tea plants [37]. We know little about the function of the CsAMT2 subfamily transporters through NH_4_^+^ absorption and utilization compared to other crops. In addition, it is unclear whether the transcriptional regulation of CsAMTs is related to NH_4_^+^ uptake in different cultivars. We hypothesized that there are several unverified AMT2-type proteins in tea plants, which would potentially regulate the NH_4_^+^ transmembrane from roots to shoots, and the regulation effects are varied in different cultivars. Given the lack of existing information, in this study, we identified five CsAMT2 subfamily members based on the latest tea plant genome database [38,39,40,41]. In addition, we investigated their expression profiles and their changing trends in response to NH_4_^+^ supply over time in three different cultivars. The objectives of this study were to functionally validate the genes regulating the NH_4_^+^ uptake characteristics of different cultivars. These results provide a foundation for further research into the roles of CsAMT2s in the N uptake and assimilation mechanism, allowing us to make full use of the NH_4_^+^ uptake characteristics of tea plants to determine their cultivation techniques, thus promoting N use efficiency (NUE) in practical plantation.

## 2. Results

### 2.1. Identification and Characterization of CsAMT2s

In this study, we obtained the top five proteins with the highest scores as candidate reference genes in each of the three tea plant genome databases (cultivar ‘Yukang 10′ and two genome versions of cultivar Shuchazao) [38,39,41] according to the sequence of *AtAMT2.1* (AT2G38290.1). The candidate reference genes information is listed in the Supplementary Materials, Table S1. Based on these genes, the CDS of five *CsAMT2* members were cloned from the cDNA of mixed leaves and roots of the FD cultivar and named based on the order of discovery and the similarity to other species. The CDS of *CsAMT2.1*, *CsAMT2.2*, *CsAMT2.3*, *CsAMT2.4*, and *CsAMT2.5* encoded 475, 478, 472, 485, and 469 amino acids, respectively. The gene sequences were basically the same as the genome data, with only individual base or amino acid differences, and the detailed sequence information has been submitted to the GenBank database; the accession numbers of *CsAMT2.1* to *CsAMT2.5* are MW751970 to MW751974, respectively.

**Figure 1:**
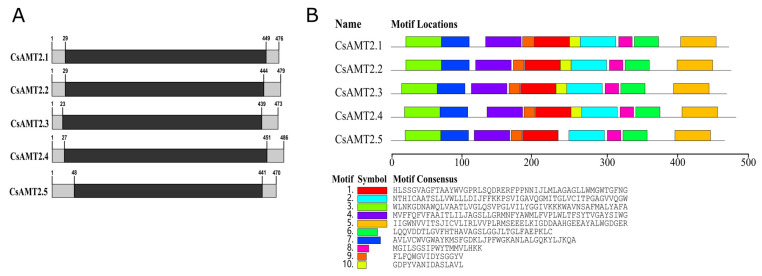
Predicted distribution of conserved domains and motifs of CsAMT2 proteins. (**A**). Ammonium_transp (PF00909) protein domains were identified by CDD database; the black boxes indicate the distribution of this conserved domain. (**B**) Positions of ten conserved motifs were identified using MEME; the different boxes and colors identify the putative motifs.

The predicted molecular weights of CsAMT2 proteins ranged from 51052.80 Da (CsAMT2.1) to 52467.24 Da (CsAMT2.4), and the theoretical pI values were from 6.06 (CsAMT2.2) to 9.12 (CsAMT2.5). The CsAMT2 proteins were stable, and the GRAVY values varied from 0.455 (CsAMT2.5) to 0.572 (CsAMT2.2), with a high content of aliphatic amino acids, indicating that they were hydrophobic proteins. Meanwhile, the CsAMT2 proteins were non-secreted proteins without signal peptides. Furthermore, subcellular localization predictions indicated that these five proteins were all located on the plasma membrane with eleven transmembrane domains (Table 1). In addition, we analyzed the distribution of the conserved domains and motifs of these five proteins in the NCBI CDD database. The results show that the CsAMT2 proteins all contained the Ammonium_transp (PF00909) structural domain (Figure 1A), which belongs to the cl03012 protein superfamily and has the property of transporting NH_4_^+^ across the membrane. CsAMT2.1–2.4 had all ten motifs, while CsAMT2.5 did not have motif10 (Figure 1B).

### 2.2. Cis-Elements in the CsAMT2 Promoters

**Figure 2:**
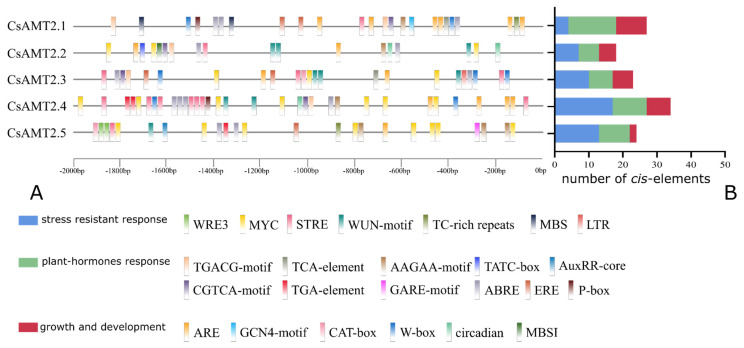
Predicted *cis*-elements in *CsAMT2* promoters. (**A**) Analysis of cis-elements in the upstream 2000 kb region of *CsAMT2* family genes in tea plants; different types of cis-elements are indicated by different colored blocks. (**B**) The three types of *cis*-elements are related to growth and development, stress resistant responsiveness, and plant hormones responsiveness.

To understand the potential regulatory mechanisms of *CsAMT2s*, the *cis*-acting regulatory elements in the promoter regions were predicted using PlantCARE. The number and names of the upstream cis-elements for five genes are listed in the Supplementary Materials, Table S2. In addition to some common elements (CAAT-box, TATA-box), there were many regulatory elements related to light signaling, stress response, hormone response, and growth and development (Figure 2). We focused on the analysis of *cis*-acting elements in the upstream sequences of *CsAMT2* genes related to growth and development, stress response, and plant hormone response. The results showed that there were seven functions related to damage defense, stress responsiveness, drought-inducibility, and low-temperature responsiveness (WUN-motif, 10, WRE3, 2, MYC, 21, STRE, 13, TC-rich repeats, 2, MBS, 2, LTR, 1) and eleven classes of regulatory elements involved in various plant hormone responses, including auxin (TGA-element, 3), abscisic acid (ABRE, 13, AAGAA-motif, 6), gibberellins (P-box, 2, GARE-motif, 1, TATC-box, 1, AuxRR-core, 1), ethylene (ERE, 5), salicylic acid (TCA-element, 2), and methyl jasmonate (TGACG-motif, 6, CGTCA-motif, 6). In addition, six elements associated with plant growth and development were predicted, mainly involved in anaerobic induction (ARE, 15), meristem development (CAT-box, 2), sucrose metabolism and stress signal regulation (W-box, 7), day–night rhythm (circadian, 2), and only one element (GCN4-motif) related to endosperm development and MBSI related to flavonoid biosynthetic genes regulation, were predicted in only *CsAMT2.1* and *CsAMT2.2*.

These *cis*-elements were distributed in the promoter regions of *CsAMT2s* widely, of which stress-resistant response elements appeared frequently, except in *CsAMT2.1*, and plant hormones elements harbored the most types. *CsAMT2.4* was predicted to have the highest total number of elements, while *CsAMT2.2* had the lowest. This indicates that the transcription levels of *CsAMT2* genes were susceptible to the influence of various environmental factors and may play a potential regulatory role in all stages of growth and development of tea plants.

### 2.3. Phylogenetic Analysis of CsAMT2 Proteins in Different Plants

**Figure 3:**
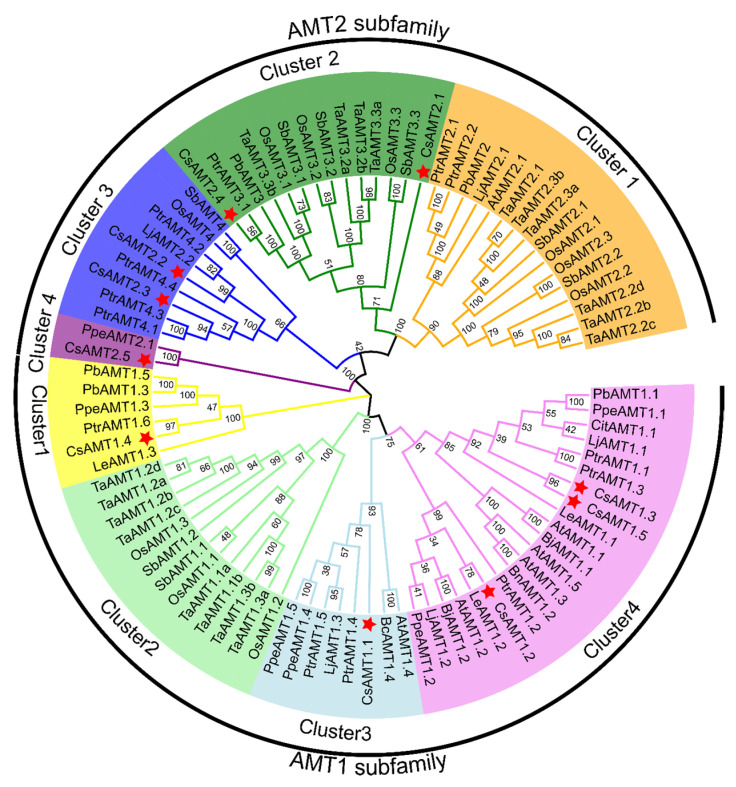
Phylogenetic analysis of proteins encoded by CsAMTs and AMTs from other plant species. The phylogenetic tree was constructed using the neighbor-joining method. Cs: *Camellia sinensis*, Os: *Oryza sativa*, Ptr: *Populus trichocarpa*, Le: *Solanum lycopersicum*, Lj: *Lotus japonicus*, Bj: *Brassica juncea*, Bc: *Brassica campestris*, Bn: *Brassica napus*, Cit: *Citrus sinensis*, Pb: *Pyrus betulaefolia*, Ppe: *Prunus persica*, Ta: *Triticum aestivum*, Sb: *Sorghum bicolor*, Vv: *Vitis vinifera*.

The determined full length amino acid sequences from 15 different species were aligned using ClustalW, and then we performed a phylogenetic analysis based on the multiple alignments results. The phylogenetic tree shows that the AMT proteins are divided into two subfamilies, and each subfamily can be further divided into four clusters. Notably, all five CsAMT2 proteins of each species belong to the AMT2 subfamily and show a cluster distribution. In detail, CsAMT2.5 and PpeAMT2.1 are in the smallest clade—cluster4. CsAMT2.2 and CsAMT2.3 are classified into the same subgroup and have a close homology relationship with the PtrAMT4s subfamily genes from *Populus trichocarpa*. CsAMT2.4 is most closely related to PtrAMT3.1, and CsAMT2.1 was related to the TaAMT3 proteins from *Triticum aestivum*, all of which were attributed to cluster2 (Figure 3).

### 2.4. Determination of N Nutrient Adaptation Types in Different Cultivars

**Figure 4:**
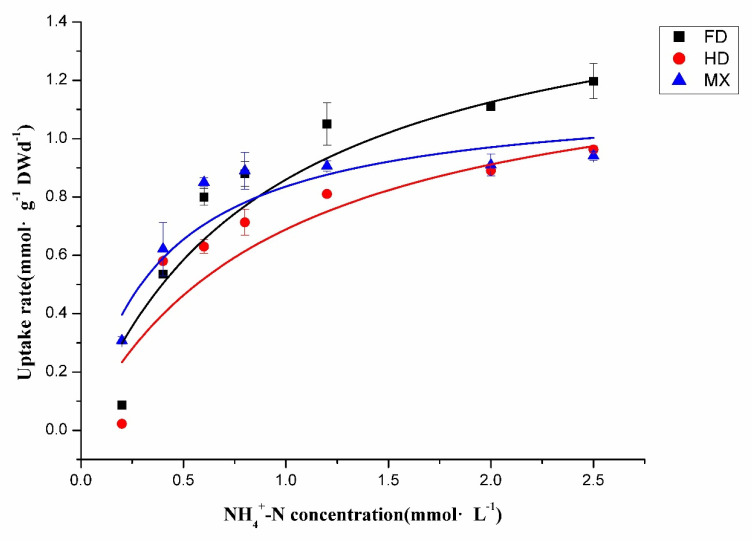
Kinetic absorption curve of NH_4_^+^–N in roots of three different tea plant cultivars. The data are presented as six biological replicates ± SD.

After short-term N starvation, the NH_4_^+^–N absorption dynamics characteristics in the roots of three different cultivars were fitted to the Michaelis–Menten equation. The NH_4_^+^–N uptake rate increased with the increase in NH_4_^+^ and tended to be saturated gradually. When the NH_4_^+^ concentration was 2.5 mmol·L^−1^, the uptake rate of the FD was higher than the other two cultivars, showing a more prominent NH_4_^+^ absorption efficiency, whereas the NH_4_^+^ uptake rates of the other two cultivars were lower (Figure 4).

The results of the NH_4_^+^–N absorption dynamics parameters show that FD had the highest V_max_ value, followed by HD, but the V_max_ of MX was below the average level. The K_m_ value of the HD and FD were more similar to one another, both approximately 2.5 times higher than MX. The α value followed the pattern of MX > FD > HD. Taken together, the FD and HD had relatively higher V_max_ values, while the MX had the highest α value, indicating its more adaptative ability to a N-deficient environment. Therefore, the NH_4_^+^ uptake characteristics of the FD had the features of high V_max_ and low K_m_, showing this cultivar has wide adaptability to soil nutrition, and we defined it as a high N efficiency (HNE) cultivar. The V_max_ and K_m_ of the HD were both relatively high, suggesting that its affinity for NH_4_^+^ is relatively low, but it was easy to obtain a higher yield in high N conditions. Hence, we considered it was a high N efficiency under high N conditions (HNEH) cultivar. MX had the lowest V_max_ and K_m_ values of these three tested cultivars, showing that this cultivar could maintain a certain NH_4_^+^ absorption efficiency under low N conditions, and we defined it as a high N efficiency under low N conditions cultivar (HNEL) (Table 2).

### 2.5. Tissue-Specific Expression Patterns of CsAMT2s

**Figure 5:**
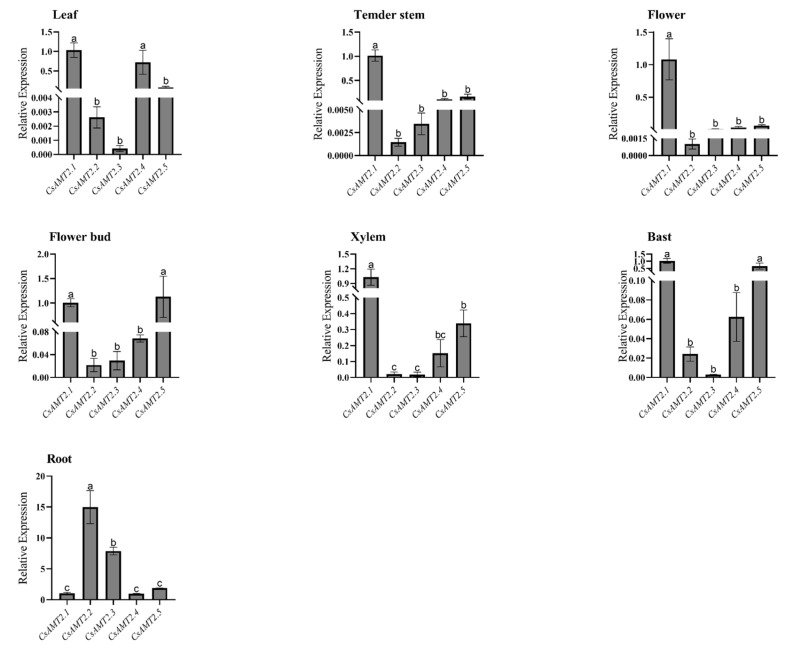
The expression of different *CsAMT2* genes in the same tissue of FD. Expressions of *CsAMT2.1* in FD were used as the control in the comparison of each tissue. The lowercase letters represent significant differences at *p* < 0.05 by using one-way analysis of variance (ANOVA) by Duncan. Three biological replicates and three technological replicates were performed for each tissue.

To clarify the potential roles of *CsAMT2* genes involved in the process of NH_4_^+^ absorption and transportation, we compared the expression of each at the same tissue. The results show that *CsAMT2.1* had higher expression levels in all tissues except the roots. Especially in the tender stems, the *CsAMT2.1* expression was significantly higher than the other four. *CsAMT2.4* was highly expressed in the leaves, and the expression of *CsAMT2.5* in the flower buds and bast was equivalent to that of *CsAMT2.1*; both were significantly higher than the other genes. It is noteworthy that the expression of *CsAMT2.2* was fifteen-fold and that of *CsAMT2.3* was seven-fold higher than those of the other three genes in the roots of tea plants (Figure 5). Upon further analysis, it was determined that the expressions of both *CsAMT2.2* and *CsAMT2.3* were significantly higher than those of previously reported AMT1 genes in tea plants [42]. The expression levels of *CsAMT2.2* were more than three times higher than those of all the other *CsAMT* genes (Supplementary Materials, Figure S1). The results imply that *CsAMT2.2* and *CsAMT2.3* might play roles in NH_4_^+^ absorption.

### 2.6. Expression Profiles of CsAMTs in Response to Different NH_4_^+^ Resupply Concentrations

**Figure 6:**
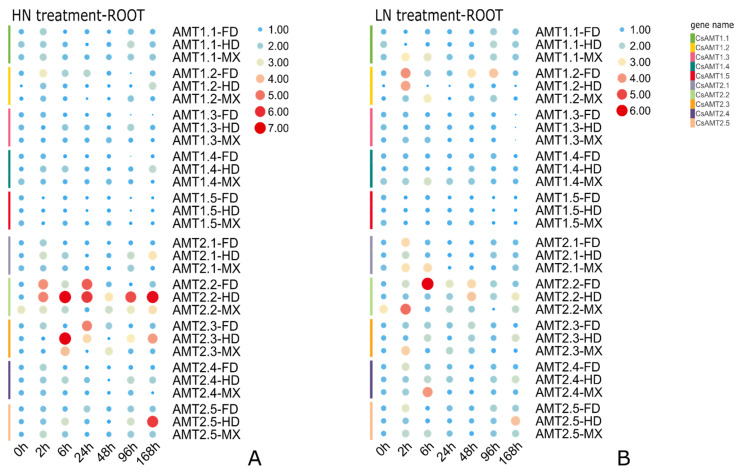
The expression diversity of each *CsAMT* gene in roots among three different tea plant cultivars. (**A**) Expression differences of each *CsAMT2* among three cultivars under high nitrogen (HN, 9.0 mmol NH_4_^+^) treatment; (**B**) Expression differences of each *CsAMT2* among three cultivars under low nitrogen (LN, 0.8 mmol NH_4_^+^) treatment. The expression level of each gene at 0 h in FD’s roots was used as the control. The average values of three biological replicates and three technological replicates were used to generate a heat map using TBtools software. The color scale represents the normalized value converted by log2. Red indicates a relatively high expression level, blue indicates a relatively low expression level, and the circle size represents the expression levels.

**Figure 7:**
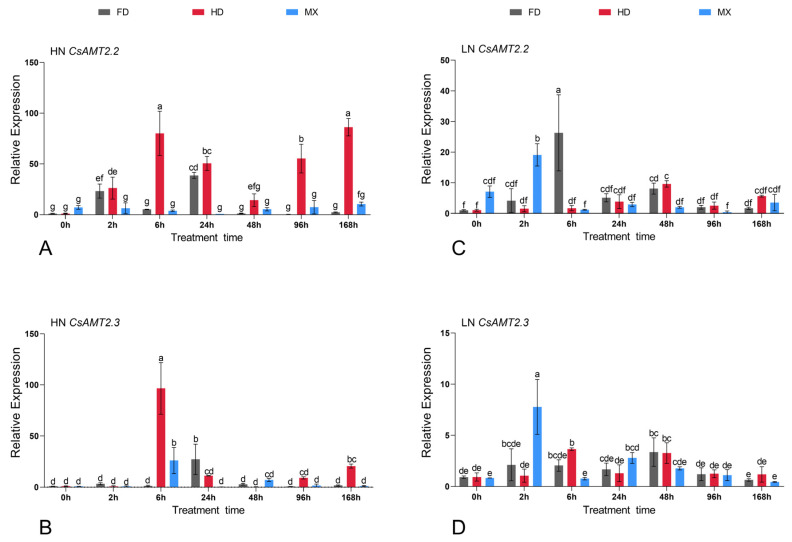
The temporal expression profiles of *CsAMT2.2* (**A**,**C**) and *CsAMT2.3* (**B**,**D**) in roots in response to high and low NH_4_^+^ resupply treatments. Data shown are the means of three biological replicates ± standard deviation (SD). By using one-way analysis of variance (ANOVA) by Duncan, the lowercase letters represent significant differences at *p* < 0.05.

To explore the role of *CsAMT2* genes among these three cultivars, we compared the expression patterns of all ten *CsAMTs* genes under HN and LN treatments. Under high NH_4_^+^ treatment, the expression levels of most *CsAMT1* genes showed an “up–down” trend, except for *CsAMT1.5*, which showed a decreasing trend after 2 h in three cultivars. It is obvious that the expression levels of *CsAMT2.2* and *CsAMT2.3* were upregulated after HN treatment (Figure 6A). Among them, *CsAMT2.2* showed induction at 2 h in the FD and HD. However, unlike the induction of *CsAMT2.2* that occurred constantly in the treated HD’s roots, *CsAMT2.2* expression decreased after 48 h of treatment in the roots of FD. In contrast, the expression of *CsAMT2.2* in the roots of the HEL cultivar MX was relatively stable. *CsAMT2.3* expression steeply increased at 6 h in the HD, and there was a second induction at 168 h, but it was induced slightly in the MX. Meanwhile the expression of *CsAMT2.3* reached its highest level at 24 h in the FD. (Figure 7A,B). In addition, the expressions of *CsAMT2.1* and *CsAMT2.5* in the HD’s roots were induced only at the end of the time series (Figure 6A).

Upon treatment with the LN condition, temporal expression of *CsAMT1.1* varied in different cultivars; it was induced in MX after 2 h LN treatment, while in FD and HD, the expression levels of *CsAMT1.1* decreased at this time point, and then showed a gradual escalation during this treatment. In addition, the expression of *CsAMT1.2* was upregulated and peaked at 2 h in the FD and HD, and showed an “up–down” trend in the HD and MX (Figure 6B). Notably, the *CsAMT2.2* expression was induced to present the significant upregulation at 6 h and 48 h in the FD and HD, respectively. Meanwhile, the expression of CsAMT2.3 showed peak induction at 48 h in these two cultivars. However, in the MX, *CsAMT2.2* and *CsAMT2.3* showed upregulation in response to the LN treatment after a short time of LN treatment at 2 h. At 0 h, the stage of N starvation, *CsAMT2.2* already had a higher relative expression in the roots of the MX (Figure 7C,D).

Taken together, these results show that the expressions of *CsAMTs* genes, especially *CsAMT2.2* and *CsAMT2.3*, differ in these three different N adaptation-type cultivars. In the FD and HD, the *CsAMT2.2* and *CsAMT2.3* genes were induced easily in a short time to the HN condition, whereas the response time of the HNEL cultivar to LN supply was faster than the other two cultivars.

### 2.7. Functional Analysis of CsAMT2.2 and CsAMT2.3 in Yeast

**Figure 8:**
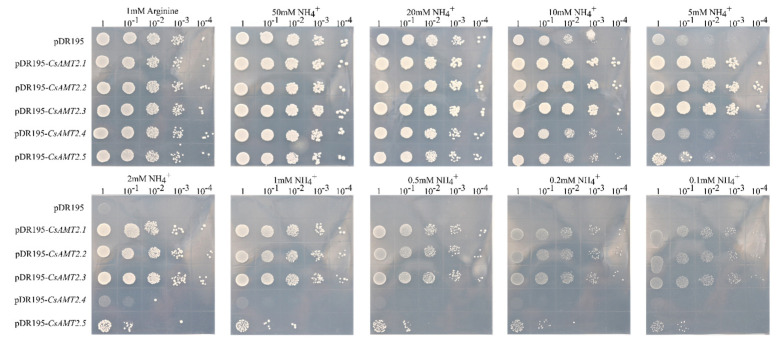
Functional growth complementation analysis of CsAMT2s in ammonium-uptake-defective yeast mutant.

The yeast mutant strain 31019b, which grows poorly at a < 5 mM NH_4_^+^ concentration as the sole N source, was used to further investigate their transport function [43]. In contrast to CK, yeast transformants carrying any CsAMT2s grew better under 50 mM, 20 mM, 10 mM, 5 mM, and 2 mM NH_4_^+^ concentrations, thus indicating that these five proteins all had the biological function of transporting NH_4_^+^ across membranes (Figure 8). However, when the exogenous NH_4_^+^ concentration was decreased to less than 1 mM, only the transformants harboring CsAMT2.1, CsAMT2.2, and CsAMT2.3 grew normally.

**Figure 9:**
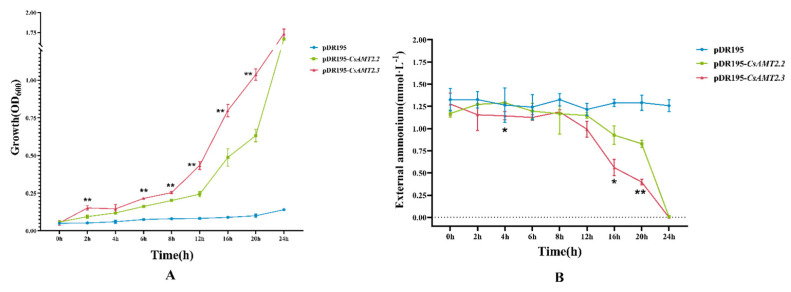
CsAMT2.2 and CsAMT2.3 mediates NH_4_^+^ retrieval in 31019b yeast mutant strain. (**A**) The growth status (OD_600_ changes) of each yeast strain over time. (**B**) The NH_4_^+^ concentration changes in the supernatant of each yeast strain over time. Three biological replicates and three technological replicates were performed for each time point. The significance analyses of *pDR195*-*CsAMT2.2* and *pDR195*-*CsAMT2.3* were performed at each time point. * indicates that the difference is significant at the 0.05 level, and ** indicates the difference is significant at the 0.01 level.

For further understanding of the NH_4_^+^ transportation efficiency of CsAMT2.2 and CsAMT2.3, we also detected the yeast cell growth status and the NH_4_^+^ changes in the culture medium supernatant over time. The yeast that expressed *CsAMT2.2* and *CsAMT2.3* grew rapidly after 8 h of transference, and the NH_4_^+^ concentration in the supernatant was also reduced greatly compared to the CK (Figure 9A). Additionally, the yeast growth rate of *pDR195*-*CsAMT2.3* was faster than that of the strains expressing *CsAMT2.2*, and the difference reached a significant level after 8 h cultivation. Furthermore, at 16 h, the yeast strains expressing *CsAMT2.3* transported more NH_4_^+^ than *CsAMT2.2*, and the concentration of NH_4_^+^ in the *pDR195*-*CsAMT2.3* medium was much lower than that of *CsAMT2.2*. Consequently, these results provide evidence that the CsAMT2.3 protein has a higher transport efficiency of NH_4_^+^ than CsAMT2.2 (Figure 9B).

## 3. Discussion

NH_4_^+^ is the most prominent from of N taken up by tea plants, as well as in the translocation and allocation of N throughout the plant. This physiological process is mainly performed by the transporters, AMTs. AMT proteins can be subdivided into two subfamilies—AMT1 and AMT2—based on amino acid sequence similarity. Five members of the *CsAMT1* subfamily were previously isolated from tea plants [42]. Here, we obtained five AMT2-type transporters in tea plants, of which CsAMT2.1 was identical to the previously published protein, CsAMT3.1 [37], and the remaining four are newly identified proteins named CsAMT2.2–CsAMT2.4.

### 3.1. N Adaptation Types Varied in Different Cultivars of Tea Plants

Increasing evidence indicates that the N uptake characteristics are varied among different cultivars of tea plants [2,37]. Previous studies have determined the N uptake efficiency by comparing the increased biomass, root morphological traits, and N accumulation under different N concentration treatments [44,45,46,47]. Since tea plants have the distinct physiological characteristics of preferentially absorbing NH_4_^+^–N [1,11], studies have tended to define the properties of N absorption by establishing a NH_4_^+^ kinetic model. This Michaelis–Menten model describes carrier-mediated ion transport with two kinetic parameters: V_max_ and K_m_. V_max_ is the maximal uptake rate, and α (V_max_/K_m_) represents the slope of the initial linear part of the curve, which is better for understanding the NH_4_^+^ capability at a low N concentration. Liu [48] showed that the V_max_ and α value were both higher in ZhongCha302, indicating this cultivar had a much strong adaptability to soil N nutrients. Longjing43, Fudingdabaicha, and Teiguanyin with high V_max_, were suitable to grow in the environment with sufficient N fertilizer, while Jiaming#1 was more resistant to barren soil compared to other cultivars as it had a lower V_max_ value and the highest α value. Thus, in tea plants, it is obvious that different cultivars possess their own adaptability to N nutrients in the soil. In our study, the results indicate that the HNE cultivar, FD, had the highest V_max_ value, suggesting its NH_4_^+^ uptake efficiency was much stronger, and that the main roots of FD might be longer than those of the other two (Supplementary Materials, Figure S2). The V_max_ of the HD were comparatively higher, indicating this HNEH cultivar may prefer higher N levels. Meanwhile, the highest α value shows that MX was the HNEL cultivar; it was more tolerant to impoverished soil, which also corresponds to the phenotype of its large number of absorbing roots (Supplementary Materials, Figure S2).

### 3.2. Tissue-Specific Expression Patterns of CsAMT2 Subfamily Genes

Numerous studies have found that AMT2-type genes are expressed in several tissues, thus causing NH_4_^+^ to be moved from intracellular production sites to consumption sites. For instance, *PbAMT2* is expressed highly in the roots, while the expression of *PbAMT3* is restricted to leaves [24]. Moreover, in *Populus trichocarpa*, a perennial woody plant, *PtrAMT2.1*, *PtrAMT2.2*, and *PtrAMT3.1* were exclusively expressed in the leaves, petioles, and senescing poplar leaves [23]. Here, *CsAMT2.1* was expressed in almost all the major organs in the tea plants, which is consistent with *AtAMT2.1* [29] and *LjAMT2.1* [25], and we speculate that it plays a role in translocating NH_4_^+^ to the aerial parts of the plant. *CsAMT2.4* was mainly expressed in the leaves, and this result echoes previous findings on *CsAMT3.1* that was cloned from Longjing43 [37]; these two proteins have only individual amino acid differences. It is noteworthy that the expression level of *CsAMT2.2* in the roots was about 15-fold higher than the other genes, including the reported key genes controlling N uptake—*CsAMT1.2*, followed by *CsAMT2.3* (Figure 5). This result suggests that the *CsAMT2.2* and *CsAMT2.3* proteins may confer unique functions in the process of NH_4_^+^ uptake by roots.

### 3.3. CsAMT2.2 and CsAMT2.3 Play Roles of NH_4_^+^ Absorption in Different Cultivars

Perennial woody plants need to adapt to the changes in nutrients in the environment over a much longer survival time scale than annual plants. Studies have shown that AMT genes are involved in responses to external NH_4_^+^–N acquisition and adaptations to different environments by regulating their own substances’ biosynthesis [29,49]. *OsAMT1.1*, *OsAMT1.2*, and *OsAMT2.2* in two different rice cultivars—Guidan4 and Nipponbare—have different transcriptional regulation modes [30]. Similarly, the expression responses to NH_4_^+^ of *OsAMT1.1* in two cultivars with different N requirements were dissimilar and its transgenic lines of biomass growth of different cultivars also had differences [31,32,50]. In spite of this, it is widely thought that the transcription levels of AMT genes could reflect their ability to absorb and assimilate N nutrients to a certain extent. In tea plants, previous studies have found that the expression of *CsAMT1.2* in the roots can be induced by low and normal N treatments over a short time in the cultivar of Zhongcha302, with a high V_max_ value [37]. The preference for NH_4_^+^ acquisition depends upon a wide range of physiological and environmental factors. Such AMT proteins have a regulatory role in the optimization and adaptation to environmental selection. For example, *MtAMT4.2* and *MtAMT4.3* in the roots of NH_4_^+^-loving perennial fruit trees—*Malus hupehensis* Rehd., were upregulated after 2 h LN treatment [28]. In our study, *CsAMT1.2* showed an “up–down–up” trend in the roots of three tested cultivars, and this increasing range was more significant under LN treatment; the results once again proved that the high-affinity transporter *CsAMT1.2* controls NH_4_^+^ uptake in the roots of tea plants. In addition, the expression levels of most *CsAMT2* genes responded more rapidly under LN condition. Even though *CsAMT2.1* had a constitutive expression pattern, the upregulated expression in the roots was observed in NHE cultivar FD and NHEL cultivar MX after short-term LN treatment. The results are similar to *PbAMT2* in *Pyrus betulaefolia* [24]; Thus, we speculate it might work at a whole plant level from the roots to the leaves and exert its N remobilization function under N-deficient conditions, and this regulation was more obvious in cultivars with high NH_4_^+^ affinity. In contrast, the NH_4_^+^ affinity of *CsAMT2.5* was much than lower than *CsAMT2.1–2.3* (Figure 8), and its temporal expression was induced significantly after 168 h treatment in the roots of HNEH cultivar HD, suggesting it might play a role in NH_4_^+^ uptake only when the external N level is stable. Remarkably, our experimental results show that the expression levels of *CsAMT2.2* and *CsAMT2.3* in the FD and HD, which were more suitable for the HN condition, increased in a short time, suggesting the strong adaptability to an HN environment of the HNE and HNEH cultivar. Meanwhile, this change was not obvious in the HNEL cultivar—MX. Under the LN treatment, these two genes were greatly induced in the MX compared to the other two cultivars, in accordance with its N deficiency tolerance capability (Figure 6). Hence, we speculate that *CsAMT2.2* and *CsAMT2.3* might regulate the NH_4_^+^ influx in roots and result in the various N adaptability in different cultivars as expected.

On the other hand, both the adapted substrate affinities and transcriptional regulation of CsAMTs make tea plants respond differently to varying nutritional conditions in the environment [51]. There are two types of NH_4_^+^ transport systems mediated by AMTs in plants: high-affinity transporters (HATS) and low-affinity transporters (LATS), and 1 mM NH_4_^+^ is the threshold [52]. From this research, CsAMT2.1, CsAMT2.2, and CsAMT2.3 belong to HATS, CsAMT2.4 and CsAMT2.5 belong to LATS, and the NH_4_^+^ transport efficiency of CsAMT2.3 was higher than that of CsAMT2.2 (Figure 8 and Figure 9). Therefore, by integrating the expression profiles and functional analysis of *CsAMT2s*, we speculate that CsAMT2.2 and CsAMT2.3 might play roles in the roots of different cultivars to make them better adapt to various nitrogenous environments. However, the latest research [53] found that the expression levels of *CsAMT3.1* and *CsAMT3.2* (the counterparts in our study were *CsAMT2.4* and *CsAMT2.5*) in roots were higher than other AMT2-type genes, and their expression responses to NH_4_^+^ were inconsistent with our results. This may have been caused by the differential cultivars and their growth conditions [54].

We also isolated the CDS of *CsAMT2.2* and *CsAMT2.3* from the HD and MX, and the sequencing results show that there were no differences in *CsAMT2.2* (Supplementary Materials, Figure S3). Meanwhile, the *CsAMT2.3* sequence of the HD was exactly as same as the one in the MX, but it had seven base differences compared to the FD (Supplementary Materials, Figure S4), two of which were non-synonymous variations (Supplementary Materials, Figure S5). Since the number and types of *cis*-elements in promoter regions were different from these two genes (Figure 2), we speculate that the CsAMT2.2 and CsAMT2.3 functions among these three cultivars may be influenced by both CDS mutation and the upstream *cis*-element regulatory modes. Unfortunately, the limited genotypes functional validation in the yeast heterologous system constrained our ability to resolve the details of each protein’s transport properties. Nevertheless, the expression and functional analysis in our experiment still provides the evidence that AMT2-type transporters play roles in regulating the absorption of external NH_4_^+^ by the roots among different cultivars. Indeed, the process of NH_4_^+^ transport depends on the synergistic effect of AMT family members [17,33]. So far, it is not clear what the differences are in the transport mode and route among the reported hub genes in the N metabolism network (*CsAMT1.2* and other AMT2-type proteins) [51]. Furthermore, exploration of the co-regulatory changes of AMT genes with other genes might also be instrumental for clarifying the balance and tight interaction between N and carbon metabolisms [55]. Consequently, expanding the range of experimental cultivars, especially some specific germplasm resources with high amino acid contents, will facilitate further elucidation of the molecular mechanism transport of NH_4_^+^–N by roots.

## 4. Materials and Methods

### 4.1. Plant Materials and Growth Conditions

The genotype Fudingdabaicha (FD) was used in this study for the experiments of molecular cloning and tissue expression pattern exploring. This cultivar was obtained in the autumn of 2019 from the tea germplasm resource nursery (27°13′15″ E, 119°35′0″ N) of the Tea Research Institute, Fujian Academy of Agricultural Sciences.

One-year-old cutting seedlings of Fudingdabaicha (FD), Huangdan (HD), and Maoxie (MX) with unified growth were selected for this study. The stable hydroponic system of tea plants referred to the method of Ruan et al. [56]. The composition of full-strength nutrient solution contained macronutrient elements (N, 3.0 mM; P, 0.07 mM; K, 0.6 mM; Mg, 0.67 mM; Ca, 0.53 mM) and micronutrient elements (B, 7 μM; Mn, 1 μM; Zn, 0.67 μM; Cu, 0.13 μM; Mo, 0.33 μM; Al, 70μM; Fe, 4.2 μM). The seedlings were fixed with foam board and sponge in water boxes. Each box contained 30 L of nutrient solution which was replaced every week and ventilated continuously. The pH was adjusted to 5.0 ± 0.5 with 1 mM NaOH and 1 mM HCl every day. The plants were grown in the greenhouse of which the parameters were set as: 30/20 °C (day/night), 14/10 h (light/dark), and 70% relative humidity. Finally, the seedlings were grown in the normal N (2 mM N) condition for 4 weeks, and then transferred into N-free nutrient solution for 2 weeks before being treated under different NH_4_^+^ concentrations.

### 4.2. RNA Extraction and cDNA Synthesis

The total RNA was extracted from the samples using the RNAperp Pure Plant Kit (Tiangen, Beijing, China). The quality and concentration of the RNA were detected by using 1% agarose gel electrophoresis and a NanoDrop 2000 ultra-micro spectrophotometer. The first-strand cDNA was synthesized after removing the gDNA using *TransScript*^®^ First-Strand cDNA Synthesis SuperMix (TransGen Biotech, Beijing, China).

### 4.3. Database Sequence Analysis and Molecular Cloning

To obtain the *CsAMT2* subfamily gene sequences in the tea plant genomes, previously published tea plant genome sequences for Shuchazao (*Camellia sinensis* var. sinensis) and Yukang 10 (*Camellia sinensis* var. assamica) were downloaded from the Tea Plant Information Archive (TPIA) database. (http://tpdb.shengxin.ren, accessed on 14 August 2022) [38,39,40,57] and the website at http://www.plantkingdomgdb.com/tea_tree/, accessed on 17 July 2017 [58]. The protein sequences of *AtAMT2.1* from *Arabidopsis* were downloaded from the Arabidopsis Information Resource (TAIR) database (https://www.arabidopsis.org/, accessed on 20 February 2021) and prepared as a background file for the BLASTp program. We used the local BLASTp function of BioEdit software with an E value setting of 1.0 E-100 to search the candidate *CsAMT2* subfamily genes according to *AtAMT2.1*. Meanwhile, we used ‘hmmbuild’ to build a hidden Markov model (HMM) profile according to the multiple sequence alignments of the candidate *CsAMT2* genes by using the online tool Clustal Omega (https://www.ebi.ac.uk/Tools/msa/clustalo/, accessed on accessed on 20 February 2021). In HMMER 3.0, we used ‘hmmsearch’ to confirm the members of the *CsAMT2* subfamily, and then named them depending on their homologous relationship. To further verify the accuracy of these sequences, primers used for gene cloning were designed by DNAMAN software, as shown in the Supplementary Materials, Table S3. The full-length CDS of each gene was amplified using the 2×SuperStar Plus PCR Mix (GeneStar, Beijing, China) from the FD genotype. The sequences accuracy was determined by sequencing, and it was submitted to GenBank database.

The physical and chemical parameters of the CsAMT2 proteins were analyzed using the ProtParam tool (http://web.expasy.org/protparam/, accessed on accessed on 20 February 2021). The protein subcellular localization prediction was performed using the online tool WOLF PSORT (https://wolfpsort.hgc.jp, accessed on accessed on 20 February 2021 ), and the transmembrane domains were predicted by using the TMHMM Server v.2.0 tool (http://www.cbs.dtu.dk/services/TMHMM/, accessed on accessed on 20 February 2021). The conserved domains and motifs were analyzed using NCBI CDD database (https://www.ncbi.nlm.nih.gov/Structure/cdd/wrpsb.cgi, accessed on accessed on 20 February 2021) and MEME online tools (http://meme-suite.org/tools/meme, accessed on accessed on 20 February 2021). The 2000 bp sequences upstream of the start codon of *CsAMT2s* were retrieved from the TPIA database, and then submitted to the PlantCARE database (http://bioinformatics.psb.ugent.be/webtools/plantcare/html, accessed on accessed on 20 February 2021) for the prediction of the *cis*-acting elements in the upstream sequences [59]. The predicted results were also annotated, categorized, and mapped using TBtools and Inkscape software [60]. The phylogenetic tree based on protein sequence alignment was constructed using the MEGA 7.0 software by the neighbor-joining method with 500 bootstrap replicates [61], and the retrieved sequences are listed in Supplementary Materials, Table S4.

### 4.4. Determination of NH_4_^+^ Dynamic Kinetic Characteristics

We used the modified hydroponic culture depletion method to study the kinetics of the NH_4_^+^–N absorption of three main cultivars—FD, HD, and MX—in Fujian Province [62,63]. After 4 weeks of normal growth with 2 mM N source, the roots of seedlings with similar growth status were washed with deionized water. After growth in a N-free nutrient solution for two weeks, two seedlings were inserted into a pot filled with 500 mL nutrient solution containing gradient N concentrations at 0, 0.2, 0.4, 0.6, 0.8, 1.2, 2.0, and 2.5 mM NH_4_^+^ from storage (NH_4_)_2_SO_4_ solution. The pH was adjusted to 5.0, and the seedlings were continuously aerated. The culture solution in each pot was added with 15% NH_4_^+^ content of dicyandiamide to prevent nitrification. Each pot was replenished after the water loss of transpiration by weight after 24 h. Six replicates were set in each treatment, and the solution samples were stored at 4 °C for subsequent analysis.

The NH_4_^+^ concentration of the solution sample was measured by the indophenol blue method [46]. The roots were separated from each tea plant and dried in an oven at 70 °C to a constant weight and then weighed. The NH_4_^+^–N absorption kinetic equation of the roots were fitted with the Michaelis–Menten model using Origin9.1 software, and calculated by a subsequent evaluation: V = V_max_C/(K_m_ + C), α = V_max_/K_m_, V_max_ (mmol·N·g^−1^·root·dry·wt·d^−1^) is the maximum absorption rate, C is the concentration of NH_4_^+^ in the solution, K_m_ (mmol·N·L^−1^) is the NH_4_ ^+^ concentration when the absorption rate is half of the maximum absorption rate, which represents the affinity of the root cells for NH_4_^+^, and the α value reflects the NH_4_^+^ absorption efficiency of the roots under low N conditions [51].

### 4.5. Quantitative Real-Time (qRT)-PCR Analysis

Seven tissues of the FD with normal growth conditions were harvested as leaf, tender stem, xylem, bast, flower, flower buds, and roots to study the tissue-specific expressions of the *CsAMT2s*. To explore the transcriptional regulation of *CsAMT2s* in the roots under different NH_4_^+^ concentration treatments among different cultivars, the hydroponic seedlings of FD, HD, and MX were transferred into N-free solution for 2 weeks, and then treated with 9.0 mM NH_4_^+^ (4.5 mM (NH_4_)_2_SO_4_, HN) and 0.8 mM NH_4_^+^ (0.4 mM (NH_4_)_2_SO_4_, LN) by resupplying (NH_4_)_2_SO_4_ as the only N source. The roots of each cultivar were collected after treatment for 0 h, 2 h, 6 h, 24 h, 48 h, 96 h, and 168 h, and were taken at each time point for later experiments. Specific primers of the *CsAMT2* genes for qRT-PCR were designed by Primer Premier 5 software, and *CsGAPDH1* (KA295375.1) was chosen as a housekeeping gene [64]; the primer information is shown in the Supplementary Materials, Table S5. *PrefectStart*^TM^ Green qPCR SuperMix (TransGen Biotech, Beijing, China) was used to conduct the qPCR reaction and the fluorescence information was collected in a qTOWER3 G Real-Time PCR Thermal Cycler (Analytik Jena AG, Jena, Germany). Triplicate biological and technical experiments were performed for each sample, and the relative expression levels were calculated using the 2^−△△Ct^ method.

### 4.6. Functional Validation of CsAMT2.2 and CsAMT2.3 in Yeast

The CDS of the *CsAMT2s* from the transcriptome cDNA of the FD’s roots were amplified by PCR using specific primers containing the Xho I and BamH I sites. The primers information is listed in the Supplementary Materials, Table S6. Five *pDR195*-*CsAMT2* plasmids were respectively transformed into the ammonium-uptake-deficient yeast mutant strain 31019b (*MATa ura3 mep1Δ mep2Δ::LEU2 mep3Δ::KanMX2*), which was kindly provided by Professor Yuan (College of Resources and Environmental Sciences, China Agricultural University). This yeast strain lacks three endogenous AMT genes (*mep1, mep2, mep3*) for the uptake of NH_4_^+^, and it cannot grow normally with <5 mM NH_4_^+^ as the sole N source [43]. Recombined plasmids were cultured in YNB medium with 1 mM arginine as the sole N source to OD_600_ = 1. Then, the cells were collected and resuspended in distilled water. The collected deposits were diluted 10 times to 10^−1^, 10^−2^, 10^−3^, and 10^−4^, then plated onto YNB medium with 3% glucose, and 0.1, 0.2, 0.5, 1, 2, 5, 10, 20, and 50 mM NH_4_^+^ and 25 mM MES were included to maintain the pH at 5.5. A NH_4_^+^ transportation efficiency comparison experiment was performed in YNB liquid medium with 2 mM NH_4_^+^ as the sole N source. After 0 h, 2 h, 4 h, 6 h, 8 h, 12 h, 16 h, 20 h, and 24 h of growth, the OD_600_ change of each yeast solution was measured, and the concentration of NH_4_^+^ in the supernatant was analyzed using the indophenol blue method [46]. Three biological replicates were performed at each time point, and the 31019b strain with an empty pDR195 vector was used as a control.

### 4.7. Statistical Analysis

All data were expressed as the mean and standard deviation (± SD). Significance differences were determined by one-way analysis of variance (ANOVA) at the significance level of *p* < 0.05 in SPSS 26.0 software. In Section 2.5, the expressions of *CsAMT2.1* were used as the control in the comparison of each tissue. In Section 2.6, the expressions of the same gene in three cultivars as a group and the expression levels of the FD’s roots at 0 h were used as controls. In Section 2.7, the significance analysis was performed at each time point, and 0 h was used as a control in the three different groups for comparison.

## 5. Conclusions

In summary, five ammonium transporters that belong to the CsAMT2 subfamily in tea plants have been cloned, and their characterization and phylogenetic trees were analyzed. The results of the expression analysis suggest that *CsAMT2.2* and *CsMT2.3* were specifically expressed in the roots. *CsAMT2.1* had high homology with *AtAMT2.1*, allowing ammonium to be moved from intracellular production sites to consumption sites for NH_4_^+^–N recycling. Furthermore, we found that *CsAMT2.2* and *CsMT2.3* were easy to induce by HN treatments in the roots of the HNE cultivar, FD, and HNEH cultivar, HD, which were suitable for growth in HN environment. Meanwhile, these two genes were extremely upregulated over a short time under the LN treatment in the MX, an HNEL cultivar that had the highest NH_4_^+^ affinity. In addition, we inferred that CsAMT2.2 and CsMT2.3 were high-affinity transporters and the transport efficiency of CsMT2.3 was much higher than that of CsAMT2.2. These findings suggest that CsAMT2.2 and CsMT2.3 mainly regulate the absorption of exogenous NH_4_^+^–N in roots. These results provide the basis for further research of the functional roles of CsAMT2 proteins in tea plants, and has certain guiding significance for the optimization of cultivation measures of different cultivars in actual production. Furthermore, many efforts involved in the differences in the regulatory roles of CsAMT in a wider range of tea germplasms needs to be explored, and the further investigation of the upstream regulators responsible for their differential functional roles.

## Figures and Tables

**Table 1:** Characterization of identified CsAMT2 proteins in tea plant.

Gene Name	CDS Length	Amino Acids	MW (Da)	pI	Instability Index	Aliphatic Index	GRAVY	Subcellular Localization	Number of Transmembrane Domain
*CsAMT2.1*	1428	475	51,052.80	7.07	31.26	103.96	0.537	Plasma membrane	11
*CsAMT2.2*	1437	478	51,958.98	6.06	28.83	108.33	0.572	Plasma membrane	11
*CsAMT2.3*	1419	472	51,984.00	6.35	34.28	102.08	0.529	Plasma membrane	11
*CsAMT2.4*	1458	485	52,467.24	7.07	30.20	99.15	0.470	Plasma membrane	11
*CsAMT2.5*	1410	469	51,564.57	9.12	35.29	102.94	0.455	Plasma membrane	11

**Table 2:** Comparison of NH_4_^+^–N absorption dynamics parameters of roots in three different cultivars.

Cultivars	V_max_ (mmol·N·g^−1^·root·DW d^−1^)	K_m_ (mmol·N·L^−1^)	α (V_max_/K_m_)	R^2^
FD	1.628 ± 0.24	0.893 ± 0.27	1.823	0.911
HD	1.346 ± 0.35	0.953 ± 0.64	1.412	0.840
MX	1.157 ± 0.39	0.384 ± 0.13	3.013	0.873
Mean	1.377	0.743	2.083	/

Values are shown as the means ± SD of six biological replicates.

## Data Availability

All the nucleotide and protein sequences of CsAMT2s in the study are publicly available. This data can be found in NCBI database, and the accession numbers are as follows: MW751970 (CsAMT2.1), MW751971 (CsAMT2.2), MW751972 (CsAMT2.3), MW751973 (CsAMT2.4), MW751974 (CsAMT2.5).

## Supplementary Materials

The supporting information can be downloaded at: https://www.mdpi.com/article/10.3390/ijms232415661/s1.
