# Supplementary material for: Genome-Wide Identification of AMT2-Type Ammonium Transporters Reveal That CsAMT2.2 and CsAMT2.3 Potentially Regulate NH4+ Absorption among Three Different Cultivars of Camellia sinensis
Source: Int J Mol Sci. 2022 Dec 10;23(24):15661. doi: 10.3390/ijms232415661 (PMC9779401; doi:10.3390/ijms232415661)
Supplement: Supplementary file 1 [file ijms-23-15661-s001.zip › Supplementary Figures.pdf]

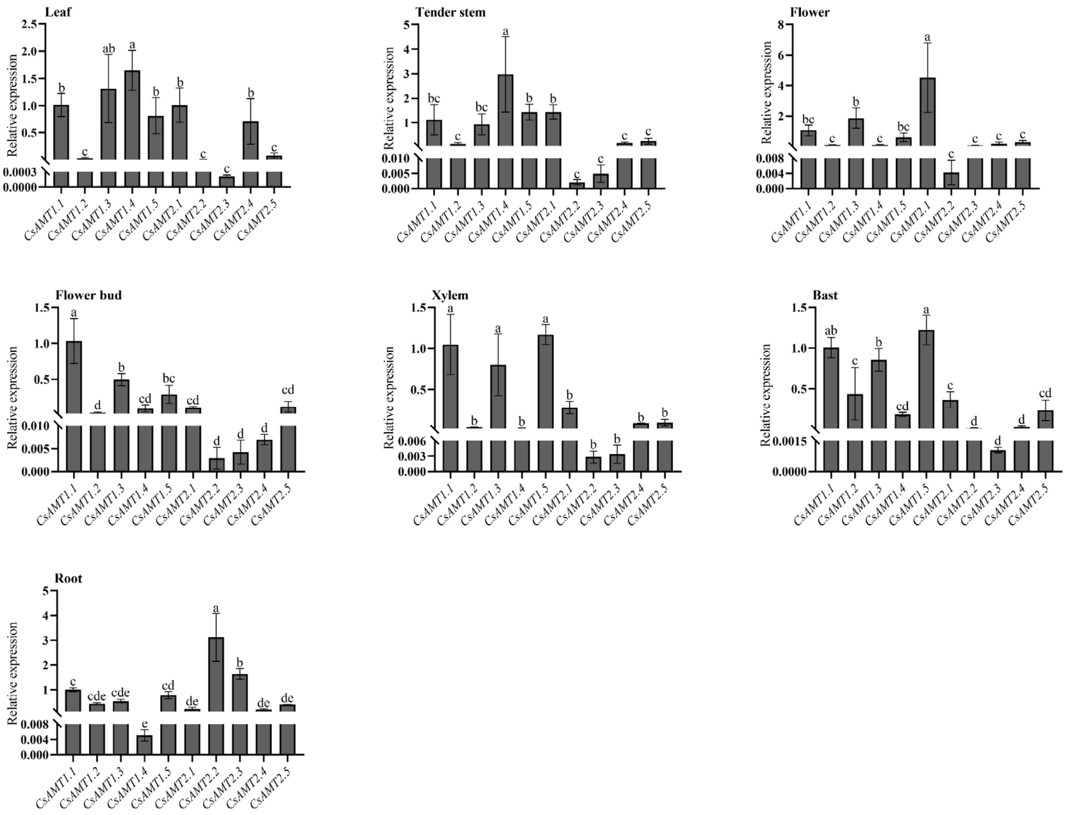


**Supplementary S1. The expression of ten *CsAMTs* genes in the same tissue of FD**

Note: Expression in *CsAMT1.1* were used as control in the comparison of each tissue, significance analysis was performed in the level of $p<0.05$. Three biological replicates and three technological replicates were performed for each tissue.

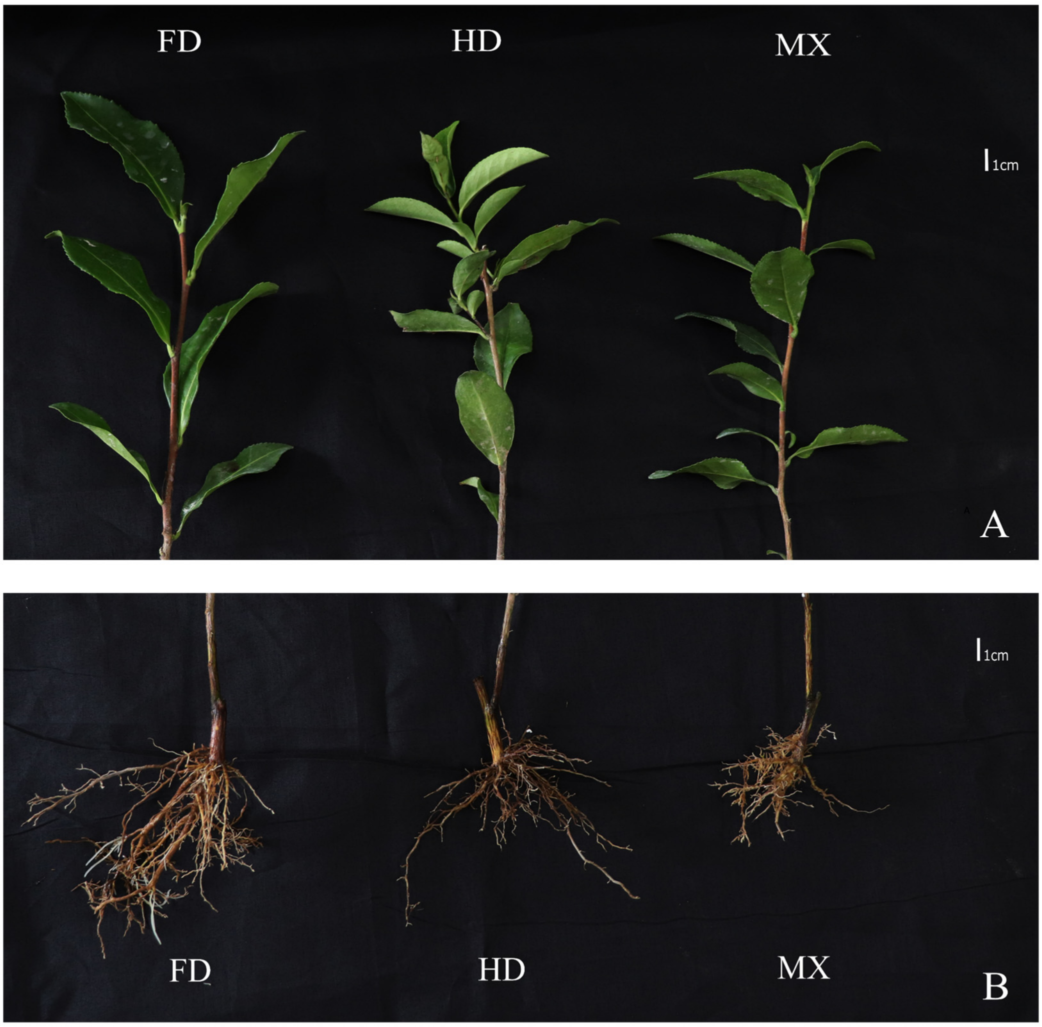


**Supplementary S2. Phenotype comparison of new shoots and roots from three different cultivars.**

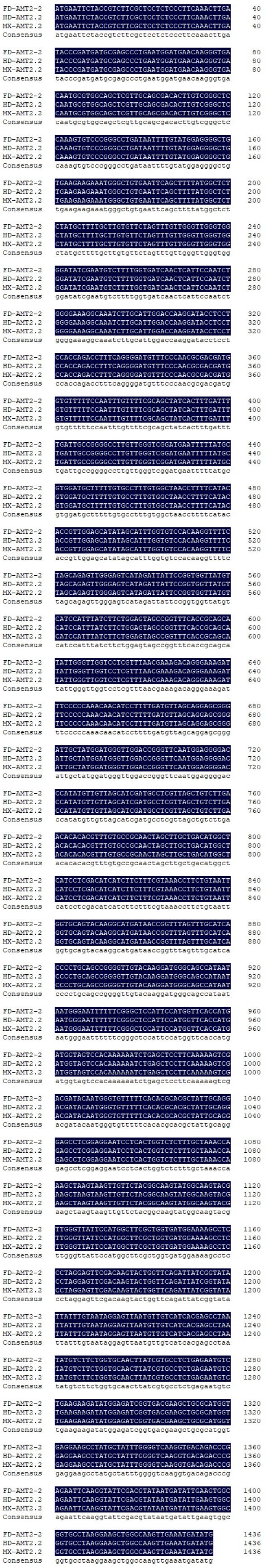

**Supplementary Figure S3. Alignment of CDS sequences of *CsAMT2.2* among FD, HD and MX.**

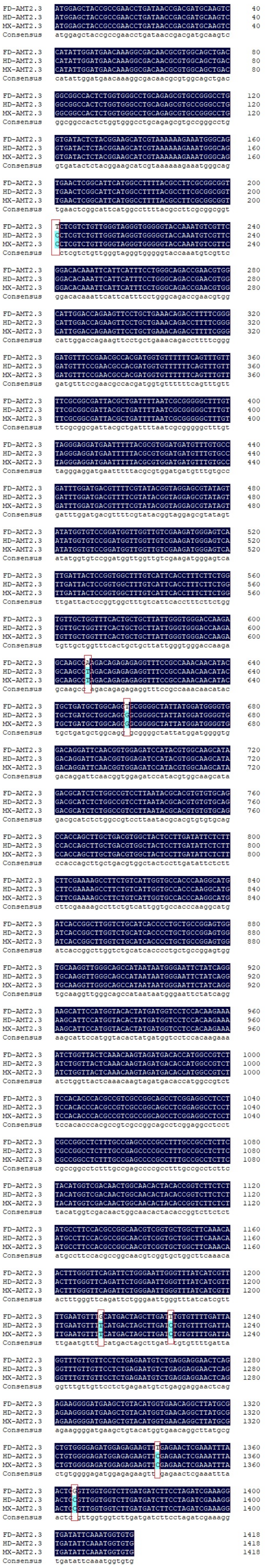

**Supplementary Figure S4. Alignment of CDS sequences of *CsAMT2.3* among FD, HD and MX.**

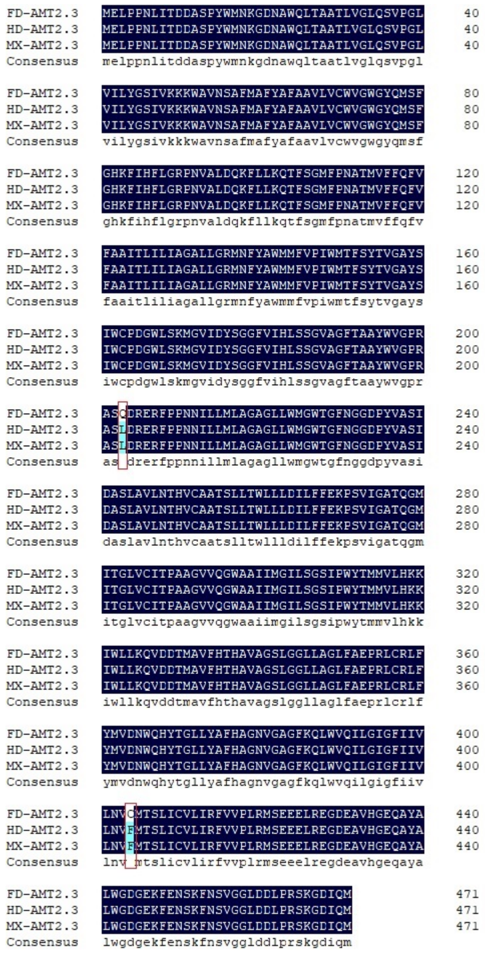


**Supplementary Figure S5. Alignment of amino acid sequences of CsAMT2.3 among FD, HD and MX.**
